# A systematic review of dietary data collection methodologies for diet diversity indicators

**DOI:** 10.3389/fnut.2024.1195799

**Published:** 2024-03-21

**Authors:** Subeg Mahal, Christopher Kucha, Ebenezer M. Kwofie, Michael Ngadi

**Affiliations:** ^1^Department of Bioresource Engineering, McGill University, Ste-Anne-de-Bellevue, QC, Canada; ^2^Department of Food Science and Technology, University of Georgia, Athens, GA, United States

**Keywords:** diet diversity, diet assessment, MDD-W, IYCF-MDD, HDDS, smartphone application

## Abstract

The purpose of the current study was to critically assess the gaps in the existing methodologies of dietary data collection for diet diversity indicators. The study proposed the importance of smartphone application to overcome the drawbacks. The review paper identified and assessed the conventional methodologies used in diet diversity indicators including Minimum Dietary Diversity for Women (MDD-W), Minimum Dietary Diversity of Infant and Young Child Feeding Practices (IYCF-MDD), and Household Dietary Diversity Score (HDDS). The 80 research studies from 38 countries were critically assessed on the basis of their research aim, study design, target audience, dietary data collection methodology, sample size, dietary data type, dietary data collection frequency, and location point of dietary data collection. Results indicated that most studies employed interviewer-administered 24-h recall assessing the dietary diversity. The review paper concluded that smartphone application had potential to overcome the identified limitations of conventional methodologies including recall bias, social-desirability bias, interviewer training, and cost–time constraints.

## 1 Introduction

Micronutrient malnutrition arises when individuals lack diet diversity and diet quality, despite having sufficient energy intake (1). An individual’s physical health, psychological health, and working capacity are correlated with nutrition status. Maintaining good health in women of reproductive age is important not only for themselves, but also for the development, growth, and long-term health of their children. Fetal development, growth, brain development, and survival rate can all be improved by adequate nutrition during the first 1000 days of a child’s life (2). Child development is vulnerable between the age of 6 and 24 months, as it involves the transition in child feeding practices from exclusive breastfeeding to the consumption of complementary foods (3). Low protein and carbohydrate diets would make women chronically malnourished mothers with a higher risk of infant mortality. Additionally, households may lack access to nutritionally adequate food during the times of food scarcity, resulting in decreased nutrient intake and diet diversity among all members of household (4). Children under the age of 5, and adults over the age of 60 are particularly sensitive to the negative effects of a poor diet (5). Diets containing a little amount of fruits, vegetables, and animal origin products, put them at greater risk of micronutrient insufficiency (6). Many households worldwide, even those with the means to eat better, consume a diet high in carbohydrates and low in nutrient-rich foods, resulting in malnutrition (7). Diet quality is a term that is often used for referring to nutrient adequacy. Diet diversity is one of the key features of diet quality (8). Diets that include a variety of food groups are critical for resisting malnutrition and foster better health in individuals and their offspring (9).

Deficits and differences in nutrition consistency at individual and household level have been known for a long time. Around 2 billion people worldwide suffer from micronutrient deficiencies, a large portion of which is attributed to monotonous diets comprising of nutrient-deficit staple crops (10, 11). As a result, the number of programmatic interventions that aim at improving diet diversity and nutrition has increased with time, as has the demand for indicators that track their impact and progress (12). Although a variety of diet diversity indicators have been developed and implemented in both research and programmatic contexts, only a few indicators have been established for use at population level in resource-poor settings. These indicators include Minimum Dietary Diversity for Women (MDD-W), Minimum Dietary Diversity of Infant and Young Child Feeding Practices (IYCF-MDD), and Household Dietary Diversity Score (HDDS). MDD-W is a dichotomous indicator of whether women 15–49 years of age have consumed at least five out of 10 defined food groups in last 24-h. The 10 defined food groups are: Grains, Roots, and Tubers; Pulses; Nuts and Seeds; Dairy; Meat, Poultry, Fish; Eggs; Dark green leafy vegetables; Other Vitamin A-rich fruits and vegetables; Other fruits; Other vegetables (13). Minimum dietary diversity (MDD) is one of the eight core indicators of Infant and Young Child Feeding Practices (IYCF) (14). MDD is defined as whether children aged between 6 and 23 months have consumed at least five out of eight defined food groups over the period of last 24-h. The eight food groups are: Breast milk; Grains, roots and tubers; Legumes and nuts; Dairy products (milk, yogurt, cheese); Flesh foods (meat, fish, poultry and liver/organ meats); Eggs; Vitamin-A rich fruits and vegetables; and Other fruits and vegetables. The proportion of women 15–49 years of age and children 6–23 months of age, who achieve this threshold in a population, can be used as a proxy indicator for higher micronutrient adequacy, one important dimension of diet quality (13). On the other hand, HDDS is an attractive proxy indicator of diet diversity representing the entire household. It computes the diet diversity score by aggregating different food groups, out of 12, consumed by all members of household over a 24-h period. Traditionally, diet diversity indicator’s dietary data is collected by written or orally reported methods from a female member or household head by employing interviewer-administered questionnaires. These questionnaires can be open recall-based or list-based (8). In open recall, interviewer asks respondent to recall all food items/ beverages consumed in the last 24 h and categorizes different constituents in their respective food groups on the questionnaire. Open recall-based questionnaires are usually administered by the multiple-pass method for 24-h recalls. The multiple-pass method consists of five steps that are followed in chronological order: quick listing of food, recalling forgotten foods, asking time and occasion of consumption, a thorough analysis of food composition, and ultimately a final review of all food items (15). On the other hand, in list-based method, the interviewer pre-defines a list of food items within each food group, and the respondent simply responds “yes” or “no” after listening to the list (16).

However, the dietary data collection methodology used traditionally has a range of drawbacks, such as respondent and researcher burden (17). The precision of 24-h recalls is hampered by memory and attention (18). Additionally, the success of method depends on persistence of the interviewer. Interviewers need to identify food ingredients and categorize them into appropriate food groups. Hiring and training educated enumerators for conducting 24-h recalls is a costly process (19) that is challenging in resource-constrained environments. Moreover, it has been confirmed that using 24-h recalls as the sole method of diet assessment results in systemic negative bias. The bias consequently leads to a significant decrease in average daily energy and nutrient intake in rural populations (20). Respondents with unstructured eating habits and regular snacking are more likely to under-report their diets (21). The feeding of 24-h recall questionnaires on a computer for analysis requires expertise and can be a time-consuming chore (22). The time and resources necessary for an interviewer-administered 24-h recall have limited its application for dietary assessment at national and subnational levels (18).

To overcome these gaps, smartphone applications can be employed as a substitute for conventional interviewer-administered 24-h recalls (23). According to Statista, there are currently 3.8 billion mobile users worldwide, which equates to 48.33 per cent of the global population. With time, smartphone capabilities have advanced, allowing them to link with the internet and run a complete operating system. Smartphone applications that enable users to track their food and beverage intake can be an easy and cost-effective way to conduct a dietary assessment (23). Smartphones not only capture food entries faster than traditional methods but also collect real-time data and substantially reduce the researcher burden (24). The ‘Eat and Track’ (EaT) (23), ‘My Meal Mate’ (24), ‘Electronic Dietary Intake Assessment’ (25, 26), ‘Easy Diet Diary’ (27), and ‘Electronic Carnet Alimentaire’ (e-CA) (28) are few dietary tracking mobile applications that have been validated with 24-h dietary recall as a reference process. Among these studies, ‘My Meal Mate’, ‘Easy Diet Diary’, and ‘Electronic Carnet Alimentaire’ (e-CA) had 72, 62.5, and 62% of participants as women, respectively (24, 26, 28).

To the best of our knowledge, this is the first study that examines the existing methodologies of diet diversity indicator’s and proposes the importance of replacing traditional methods with a smartphone application. The findings of this review paper helped us to identify and analyze the potential gaps in traditional methodologies. In the second stage, we propose that using a smartphone application for diet diversity indicators to capture and analyze data in real-time would help in overcoming the constraints of traditional methods, while improve the quality of data collection by increasing efficiency and limiting the misreporting errors.

## 2 Materials and methods

### 2.1 Literature search

The goal of the literature search was to identify and assess the methodologies employed in studies that implement MDD-W, IYCF-MDD, and HDDS as diet diversity indicator for women, children, and households, respectively. Relevant literature includes the FAO report “Moving forward on choosing a standard operational indicator of women’s dietary diversity” (29), the “Nutrition baseline survey summary report” (30) and systematic reviews of research on nutrition-sensitive agriculture that aided in the development of search strategy (31–34). Keywords search in Scopus, MDPI Nutrients, Web of Science, PubMed, ScienceDirect, Agris (a literature search portal of the United Nations Food and Agriculture Organization), and Google Scholar was conducted in May 2021 to include peer-reviewed studies published in English. The keywords employed in the literature search were “women,” “children,” “households,” “MDD-W,” “MDD,” “HDDS,” “nutrition-sensitive interventions,” “dietary diversity,” “dietary quality,” “food consumption,” “food variety,” “24-h dietary recall,” and “food frequency questionnaire.” The literature search was carried out over a time period of 11 months. This review considered all types of research designs related to diet diversity indicators, ranging from cross-sectional to cohort studies, as well as other impact evaluation or intervention studies.

### 2.2 Data screening and classification

All research papers were screened twice. In the initial screening stage, titles and abstracts were reviewed, and studies unrelated to the evaluation process were excluded. This was followed by a comprehensive text screening to ensure that studies met the second-stage eligibility criteria: studies that scrutinized, evaluated, associated, or validated either of diet diversity indicator among MDD-W, IYCF-MDD, and HDDS, with or without other household or individual diet diversity/ diet quality indicators, factors, or characteristics. The following data was tabulated to aid the full-text screening: (i) Research aim (purpose of the study); (ii) Study design (e.g., baseline survey of an intervention); (iii) Country (location of the study); (iv) Target audience [subject, e.g., pregnant women (15–49 years)]; (v) Dietary data collection methodology (e.g., 24-h dietary recall using the multiple-pass method); (vi) Sample size (number of participants involved, e.g., *N* = 558); (vii) Dietary data type (e.g., Quantitative or Qualitative); (viii) Dietary data collection frequency (number of times dietary data collected, e.g., once every year, for 3 years; (ix) Dietary data collection point (place where data was collected, e.g., household).

After screening, 80 studies were chosen to be included in this review. The applicability and methodology of these studies were assessed critically. To begin classification, studies were categorized according to methodology, whether the dietary data was gathered using an interviewer-administered recall (*n* = 78), self-administered recall (*n* = 2), or both (*n* = 0). The studies were further classified into four categories: 24-h (24-h) dietary recall, 48-h (48-h) dietary recall, 7-day (7-d) dietary recall, 30-day (30-d) dietary recall, and 1-year (1-y) dietary recall. Dietary data was classified as quantitative if the portion estimation of food was done by weighing scale, food photo atlas, or standard household utensils, including pots, plates, bowls, cups, or spoons. On the contrary, portion estimate was classed as semi-quantitative if it was performed solely to get an idea of the food quantity, else, it was categorized as qualitative. Dietary data collection frequency was classified as consecutive, if diets were recorded on sequential days, otherwise, it was classified as non-consecutive.

All critical assessment disagreements among the reviewing co-authors were settled through discussion.

## 3 Results

### 3.1 Description of the studies

The context and methodology used in the 80 research studies included in this review are summarized in Table 1. The studies have evidence from 38 countries, including one from Oceania (Fiji), two from North America (United States and Costa Rica), seven from South America (Brazil, Chile, Colombia, Ecuador, Peru, Suriname, and Venezuela), 12 from Asia (Bangladesh, Cambodia, China, India, Indonesia, Iran, Laos, Lebanon, Nepal, Pakistan, Philippines, and Sri Lanka) and 16 from Sub-Saharan Africa (Benin, Burkina Faso, Ethiopia, Ghana, Kenya, Malawi, Mali, Mozambique, Nigeria, Rwanda, Somalia, South Africa, Tanzania, Uganda, Zambia, and Zimbabwe). Seven studies present findings from multiple countries (54, 66, 73, 75, 100). In terms of their purpose, there was significant heterogeneity across 80 studies regarding association with micronutrient adequacy, household food insecurity, agricultural food production diversity, seasonal food patterns, food purchasing practices, women empowerment, antenatal care practices, maternal health care, child growth, child stunting, prevalence of anemia, and bone fractures. Two studies were designed in response to an increased demand for an indicator that can be expressed in terms of the prevalence of meeting a minimum acceptable level of diet diversity in women of reproductive age, resulting in the development of MDD-W as a dichotomous indicator (73, 75). Most of the studies were cross-sectional surveys that looked at association rather than causation. With sample sizes ranging from 40 in a pregnancy cohort study (76) to 41,101 in a prospective study (36), the number of households or individuals surveyed in these studies varied substantially.

**Table 1:** An overview of context and methodology of 80 research studies included in this review.

S. No.	Author/year	Research aim	Study design	Country	Target audience	Dietary data				
						Methodology	Type	Collection frequency	Collection point	Sample size
1	Melby et al. (2020) (35)	To establish relationship among agricultural food production diversity (FPD), Diet Diversity (DD), and household food insecurity (HFI)	Cross-sectional survey	Ecuador	Non-pregnant women (18–85 years)	24-h dietary recall Multiple pass	Quantitative	1 day	Household	*N* = 558
2	Jones et al. (2018) (36)	To establish association of farm level agricultural biodiversity with DD and Diet Quality (DQ) To assess the effect of market orientation on this association	Cross-sectional survey	Peru	Households with woman (18–49 years)	24-h dietary recall questionnaire. Multimodule household survey questionnaire	Quantitative	Non-consecutive 2 days	Household	*N* = 100
3	Zhang et al. (2020) (37)	To investigate effect of DD on any type of fracture in adults	Secondary analysis of cross-sectional survey	China	Adults (40 + years)	24-h dietary recall questionnaire Household food weight inventory	Quantitative	Consecutive 3 days	Household	*N* = 10,192 (4,795 Men; 5,397 Women)
4	Nguyen et al. (2018) (38)	-To compare micronutrient adequacy between pregnant adolescent girls and women -To check performance of Women Diet Diversity Score in predicting mean probability of adequacy -To check performance of MDD-W in pregnant adolescent girls and women	Secondary analysis of cross-sectional survey	Bangladesh	Pregnant women (18 + years)	24-h dietary recall Multiple pass 24-h dietary recall List-based questionnaire	Quantitative	Twice, same day	Household	*N* = 600
5	Seiermann et al. (2021) (39)	To check effect of dietary changes on women’s health during Ramadan	Secondary analysis of repeated cross-sectional survey	Bangladesh	Married women	24-h dietary recall	Qualitative	Once every 2 months, for 5 years	Household	*N* = 852
6	Sultana et al. (2019) (40)	To assess DD and nutrition status of residential students	Cross-sectional survey	Bangladesh	Women (18–26 years)	24-h dietary recall -Multiple pass	Quantitative	Consecutive 3 days	University	*N* = 160
7	Bellon et al. (2016) (41)	To investigate relationship of diversity of plant species raised and variety of foods purchased by women of rural households on DD under different market conditions	Cross-sectional survey	Benin	Households with women (15–49 years)	24-h dietary recall Semi-structured questionnaire Food frequency questionnaire (FFQ)	Qualitative	−15 days (Farm diversity) −7 day (Market diversity) −1 day (Diet diversity)	Household	*N* = 652
8	Diop et al. (2021) (42)	To examine performance of indicators in predicting micronutrient adequacy	Baseline survey of an intervention	Burkina Faso	Pairs of children (24–59 months old) and women	24-h dietary recall Computer assisted personal interviewing	Quantitative	Non-consecutive 2 days	Household	*N* = 1,066
9	Custodio et al. (2020) (43)	To check association of MDD-W with food security and purchasing practices	Secondary analysis of repeated Cross-sectional survey	Burkina Faso	Women (15–49 years)	24-h dietary recall	Qualitative	Once every year, for 3 years	Household	*N* = 12,754
10	Getacher et al. (2020) (44)	To determine prevalence of MDD-W and associated factors among lactating mothers	Cross-sectional survey	Ethiopia	Women (15–49 years)	24-h dietary recall	Qualitative	1 day	Household	*N* = 652
11	Hanley-Cook et al. (2020) (45)	To assess validity of List-based and Open recall-based methods with weighed food record for MDD-W	Cross-sectional survey	Cambodia Ethiopia Zambia	Women (15–49 years)	24-h dietary recall Weighed food records	Quantitative	1 day	Household	*N* = 1,337
12	Girma et al. (2019) (46)	To check nutrition and other factors that contribute to low birth weight in newborns	Cross-sectional survey	Ethiopia	Women and Newborn infants	24-h dietary recall Questionnaires on socio-economic, medical, behavior, environment, and infant related factors	Qualitative	1 day	Health care facilities	*N* = 279
13	Gyimah et al. (2021) (47)	To understand factors contributing to DD in pregnant women	Longitudinal study	Ghana	Pregnant adolescents	24-h dietary recall Questionnaire on socio-demographic factors	Quantitative	3 days	Health care facilities	*N* = 416
14	Agbozo et al. (2020) (48)	To determine link between maternal food habits, antenatal care practices and the prevalence of anemia in the first, second, and third trimesters of pregnancy	Prospective study	Ghana	Women (15–49 years)	24-h dietary recall FFQ	Qualitative	1 day	Health care facilities	*N* = 415
15	Saaka et al. (2021) (49)	To determine the independent predictors of DD and their agreement in nutrition status of pregnant women	Cross-sectional survey	Ghana	Women (15–49 years)	24-h dietary recall −7-day FFQ	Quantitative	1 day	Household	*N* = 423
16	Ayensu et al. (2020) (50)	To determine the prevalence of anemia and dietary micronutrient intakes in pregnant women	Cross-sectional survey	Ghana	Pregnant women (15–49 years)	24-h dietary recall -FFQ	Quantitative	Non-consecutive 3 days	Household	*N* = 379
17	Bukari et al. (2021) (51)	To examine diet quality and its association with household factors and gestational age	Cross-sectional survey	Ghana	Pregnant women (15–49 years)	24-h dietary recall Questionnaires on socio-demographic characteristics	Qualitative	1 day	Health care facilities	*N* = 403
18	Saaka et al. (2017) (52)	To evaluate maternal dietary intake and its association with nutrition status	Cross-sectional survey	Ghana	Households with pregnant women (15–49 years)	24-h dietary recall -FFQ	Qualitative	1 day	Household	*N* = 400
19	Gupta et al. (2019) (53)	To examine women’s agricultural empowerment and iron in-sufficiency	Agriculture-nutrition study (Cross-sectional survey)	India	Non-pregnant Non-lactating (NPNL) women (15–49 years)	24-h dietary recall -FFQ	Quantitative	1 day	Household	*N* = 960
20	Nguyen et al. (2020) (54)	To evaluate performance of list-based and quantitative open recall-based methods in predicting micronutrient adequacy	Secondary analysis of baseline survey	Bangladesh India	Pregnant women	24-h dietary recall Qualitative list-based and quantitative open-recall based questionnaire	Quantitative	1 day Repeated for 10% (Non-consecutive day)	Household	*N* = 1,255
21	Ghosh-Jerath et al. (2021) (55)	To determine link between production and access to food, DD, and nutrient intake	Cross-sectional survey	India	Households with Non-pregnant (56) woman (15–49 years) and child (6–54 months)	24-h dietary recall Multiple pass -FFQ	Quantitative	Non-consecutive 2 days	Household	*N* = 201
22	Diana et al. (2019) (56)	To analyze DD and DQ among women	Cross-sectional survey	Indonesia	Anemic pregnant women	24-h dietary recall	Quantitative	2 days	Household	*N* = 152
23	Gitagia et al. (2019) (57)	To record Agrobiodiversity, DD and factors influencing them	Cross-sectional survey	Kenya	Women (18–49 years)	24-h dietary recall Semi quantitative questionnaire	Semi-quantitative	1 day	Household	*N* = 384
24	Lamanna et al. (2019) (19)	To check degree of agreement between Computer Assisted Telephone Interviewing (CATI) and Traditional methods	Validation study	Kenya	Households with woman (15–49 years) and child (6–23 months)	24-h dietary recall -CATI	–	2 days	Household	*N* = 1,821
25	Jomaa et al. (2020) (58)	To investigate the links between household food insecurity (HFI) and mother’s sociodemographic, anthropometric, and dietary intakes	Secondary analysis of cross-sectional survey	Lebanon	Households with mother and their child (4–18 years)	24-h dietary recall	Quantitative	1 day	Household	*N* = 1,204
26	Adubra et al. (2019) (59)	To investigate agreement of MDD-W with household food insecurity and farm production diversity	Baseline survey of a 3-year intervention (Cross-sectional survey)	Mali	Households with mother–child pair	24-h dietary recall Android tablet	Qualitative	1 day	Household	*N* = 5,046
27	Dulal et al., (2017) (60)	To check association between Enhanced Homestead Food Production (EHFP) and DD of mother and child	Evaluation of ongoing intervention (Repeated cross-sectional survey)	Nepal	Mothers and children (6–23 months)	7-day dietary recall	–	2 days	Household	*N* = 3,095 (2,101 mothers; 994 children)
28	Shrestha et al. (2021) (61)	To assess DD and associated factors in hilly regions with urban municipality	Cross-sectional survey	Nepal	Pregnant women	24-h dietary recall	Semi-quantitative	1 day	Household	*N* = 327
29	Samuel et al. (2019) (62)	Comparison of DD in Cassava value chain households and non-Cassava value chain households	Descriptive cross-sectional survey	Nigeria	Households	24-h dietary recall -FFQ	–	Non-consecutive 2 days	Household	*N* = 572
30	Brazier et al. (2020) (63)	To assess DD and nutritional status of women in marginalized community	Repeated cross-sectional survey	Pakistan	Households with NPNL women (16–49 years)	24-h dietary recall Multiple pass method	Semi-quantitative	5 days	Household	*N* = 47
31	Nsereko et al. (2020) (64)	To discover modifiable risk factors for targeted interventions that aim to reduce Preterm birth	Prospective, longitudinal study	Rwanda	Pregnant women (18–49 years)	24-h dietary recall FFQ	–	1 day	University	*N* = 367
32	Chakona and Shackleton (2017) (65)	To assess DD and establish relation between MDD-W and Household characteristics	Cross-sectional survey	South Africa	Women (15–49 years)	48-h dietary recall -FFQ	–	4 days	Household	*N* = 554
33	Gómez et al. (2020) (66)	To establish relation between MDD-W and micronutrient adequacy among women from Latin American countries	Secondary analysis of cross-sectional survey	Brazil, Colombia, Costa Rica, Chile, Ecuador, Peru, Venezuela	NPNL women (15–49 years)	24-h dietary recall Multiple pass method	Quantitative	Non-consecutive 2 days	Household	*N* = 3,704
34	Weerasekara et al. (2020) (67)	To evaluate DD, nutrient adequacy, dietary and traditional food patterns in marginalized areas	Cross-sectional survey	Sri Lanka	Women (18–49 years)	24-h dietary recall Multiple pass method	Quantitative	1 day	Household	*N* = 400
35	Madzorera et al. (2020) (68)	To establish link among maternal DD, DQ with birth outcomes in pregnant women	Secondary analysis of placebo-controlled study	Tanzania	Pregnant women (18–49 years)	24-h dietary recall	Quantitative	Once in a month, for 6 months	Health care facilities	*N* = 7,553
36	Conti et al. (2021) (69)	To inspect factors contributing to micronutrient adequacy in women	Cross-sectional survey	Tanzania	Women (14–49 years)	24-h dietary recall	Quantitative	1 day	Household	*N* = 141
37	Huang et al. (2018) (70)	To recognize factors of maternal DD and its association with child growth outcomes	Longitudinal survey	Tanzania	Mother–child (under 24 months) pairs	30-day dietary recall FFQ	Qualitative	1 day	Health care facilities	*N* = 361
38	Madzorera et al. (2021) (71)	To examine role of prenatal maternal DD in infant growth	Secondary analysis of a birth cohort study	Uganda	Pregnant women (15–49 years)	24-h dietary recall	–	1 day	Household	*N* = 3,291
39	Arimond et al. (2010) (72)	To create simple indicator that can be served as proxy to measure the micronutrient of women’s diets	Secondary analysis of 24-h recalls (collected for distinct purposes)	Bangladesh, Burkina Faso, Mali, Mozambique, Philippines	Women (15–49 years)	24-h dietary recall	Quantitative	Non-consecutive 2 days	Household	*N* = 2,560
40	Yves Martin-Prevel et al. (2017) (73)	To develop dichotomous indicator for assessing DD in women of reproductive age	Secondary analysis of 24-h recalls (collected for distinct purposes)	Bangladesh Burkina Faso Mali Mozambique Philippines Uganda	Women (15–49 years)	24-h dietary recall -Multiple pass method	Quantitative	Non-consecutive 2 days	Household	*N* = 4,166
41	Ahern et al. (2021) (74)	To investigate seasonal variations in MDD-W and food group intake in regions where seasonal agricultural production and food availability have significant impact on DD	Repeated cross-sectional survey	Malawi Zambia	Women (15–49 years)	24-h dietary recall	Qualitative	Once every month, for 11 rounds	Household	*N* = 200
42	Kornatowski and Comstock (2018) (75)	To assess DD in women during their third trimester of pregnancy and establish relation with pre-pregnancy Body Mass Index	Secondary analysis of pregnancy cohort study	United States	Women (18+ years)	24-h dietary recall Self-administered questionnaires	Qualitative	1 day	Health care facilities	*N* = 40
43	Gicevic et al. (2018) (76)	To assess bonding of DD and DQ scores with gestational diabetes mellitus and hypertensive disorders of pregnancy	Prospective study	United States	Women (24–44 years)	1-year dietary recall FFQ Self-administered questionnaires	Semi-quantitative	Once every year, for 4 years	Health care facilities	*N* = 41,101
44	Paré et al. (2019) (77)	To determine the prevalence of wasting in children aged 6–23 months	Secondary analysis of Cross-sectional survey	Burkina Faso	Households with children 6–23 months of age	24-h dietary recall	Qualitative	1 day	Household	*N* = 956
45	Hipgrave et al. (2014) (78)	To assess IYCF and anemia in central and western China	Cross-sectional survey	China	Households with children 6–23 months of age	24-h dietary recall FFQ	Semi-quantitative	1 day	Household	*N* = 2,244
46	Wuneh et al. (2019) (79)	To assess Minimum Meal Frequency (MMF) and MDD	Cross-sectional survey	Ethiopia	Households with children 6–23 months of age	24-h dietary recall Open recall-based questionnaire	Qualitative	1 day	Household	*N* = 807
47	Guja et al. (2021) (80)	To examine concordance between mother and child diet diversity for designing intervention programs	Cross-sectional survey	Ethiopia	Mother and child pair (6–23 months age)	−24-h dietary recall -FFQ	Qualitative	1 day	Household	*N* = 796
48	Kim et al. (2016) (81)	To evaluate the effect of interventions on IYCF practices and anthropometry	Repeated cross-sectional survey	Ethiopia	Households with children 0–59 months of age	24-h dietary recall	Qualitative	Twice, at baseline and endline of intervention	Household	*N* = 2,969
49	Kamran et al. (2017) (82)	To assess determinants of complementary feeding practices	Cross-sectional survey	Iran	Mothers of children aged 6–23 months	24-h dietary recall -FFQ	Qualitative	1 day	Household	*N* = 576
50	Chama (2020) (83)	To determine the contribution of edible non-timber forest products to nutritional status	Cross-sectional survey	Zambia	Households with mother and child (6–23 months) pair	24-h dietary recall List-based questionnaire -FFQ	Both Qualitative and Quantitative	1 day	Household	*N* = 158
51	Modugu et al. (2022) (84)	To determine influence of gender, and migration on IYCF practices	Cross-sectional survey	India	Households with mother and child (6–23 months) pair	24-h dietary recall FFQ	Both qualitative and quantitative	1 day	Household	*N* = 325
52	Wormer et al. (2021) (85)	To determine the association between respiratory tract infections and IYCF practices	Cross-sectional survey	Suriname	Households with mother and child (12 months) pair	24-h dietary recall	Qualitative	1 day	Household	*N* = 763
53	Kogade et al. (2019) (86)	To evaluate association of IYCF practices with sociocultural determinants	Cross-sectional survey	India	Mothers of children aged 0–23 months of age	24-h dietary recall	–	1 day	Household	*N* = 612
54	Solomon et al. (2017) (87)	To assess the level of MDD and associated factors	Cross-sectional survey	Ethiopia	Mothers of children aged 6–23 months of age	24-h dietary recall	Quantitative	1 day	Health care facility	*N* = 352
55	Molla et al. (2021) (88)	To determine DD and associated factors	Cross-sectional survey	Ethiopia	Households with mother and child (6–23 months) pair	24-h dietary recall	Qualitative	1 day	Household	*N* = 665
56	Dangura (2017) (89)	To determine DD level and associated factors in Gorche district	Cross-sectional survey	Ethiopia	Households with mother and child (6–23 months) pair	24-h dietary recall	Quantitative	1 day	Household	*N* = 417
57	Mekonnen et al. (2017) (90)	To determine MMF, MDD and associated factors	Cross-sectional survey	Ethiopia	Households with mother/caregiver and child (6–23 months) pair	24-h dietary recall -FFQ	–	1 day	Household	*N* = 623
58	Belew et al. (2017) (91)	To determine MMF, MDD and associated factors	Cross-sectional survey	Ethiopia	Households with mother and child (6–23 months) pair	24-h dietary recall Open recall-based questionnaire	Qualitative	1 day	Household	*N* = 1,034
59	Gezahegn and Tegegne (2020) (92)	To determine DD and associated predictors in Gorche district	Cross-sectional survey	Ethiopia	Households with mother and child (6–23 months) pair	24-h dietary recall Open recall-based questionnaire	–	1 day	Household	*N* = 517
60	Blackstone and Sanghvi (2018) (3)	To measure maternal, household, and MDD characteristics	Secondary analysis of cross-sectional survey	Bangladesh	Households with mother and child (6–23 months) pair	24-h dietary recall	–	Twice in 4 years	Household	N_1_ = 2,925 N_2_ = 2,908
61	Zhao et al. (2021) (93)	To determine relation between dietary diversity, meal frequency, and early childhood developmental outcomes	Secondary analysis of cross-sectional survey	China	Households with mother and child (6–23 months) pair	24-h dietary recall	Qualitative	1 day	Village clinic	N = 1,534
62	Chilinda et al. (2021) (94)	To explore the effect of household water access on MDD standards	Secondary analysis of cross-sectional survey	Malawi	Households with mother and child (6–23 months) pair	24-h dietary recall	Qualitative	1 day	Household	*N* = 4,727
63	Di Marcantonio et al. (2020) (95)	To assess DD and identify the associated factors	Cross-sectional survey	Somalia	Households with mother and child (6–23 months) pair	24-h dietary recall	–	1 day	Household	*N* = 2,922
64	Rubhara et al. (2020) (96)	To assess the impact of cash crop production on household food security	Cross sectional survey	Zimbabwe	Households	24-h dietary recall	Qualitative	1 day	Household	*N* = 281
65	Gandure et al. (2010) (97)	To analyze food security indicators used to assess households	Cross sectional survey	Zimbabwe	Households	24-h dietary recall	Qualitative	1 day	Household	*N* = 178
66	Cheteni et al. (2020) (98)	To determine HDDS and food security	Cross sectional survey	South Africa	Households	7-d dietary recall	Qualitative	1 day	Household	*N* = 296
67	Kennedy et al. (2010) (99)	To provide an overview and compare the performance of HDDS and Food Consumption Score (FCS)	Cross sectional survey	Burkina Faso Lao PDR Uganda	Households	24-h dietary recall 7-d dietary recall FFQ List-based questionnaire	Qualitative	1 day	Household	*N* = 3,913
68	McDonald et al. (2015) (100)	To identify correlates of household food insecurity and poor dietary diversity	Baseline survey of an intervention	Cambodia	Households	24-h dietary recall	Quantitative	1 day	Household	*N* = 900
69	Schwei et al. (2017) (7)	To define a relationship between HDDS, food security, and consumption of Vitamin A-rich foods	Cross sectional survey	Ethiopia	Households	24-h dietary recall List-based questionnaire	Qualitative	1 day	Household	*N* = 300
70	Aweke et al. (2021) (101)	To examine the impact of agricultural technologies on household food and nutrition security	Cross sectional survey	Ethiopia	Households	24-h dietary recall 7-d dietary recall -FFQ	Quantitative	1 day	Household	*N* = 248
71	Melaku et al. (2018) (102)	To use dietary patterns as alternative to diet diversity scores, and investigate their association with childhood stunting	Cross sectional survey	Ethiopia	Households with mother child pair	24-h dietary recall	Qualitative	1 day	Household	*N* = 3,788
72	O’Meara et al. (2019) (103)	To examine diet diversity in indigenous food-producing households	Cross sectional survey	Fiji	Households	24-h dietary recall Open recall-based questionnaire	Qualitative	1 day	Household	*N* = 161
73	Mahmudiono et al. (2017) (104)	To explore the relationship between child stunting and household dietary diversity	Cross sectional survey	Indonesia	Households with children below 5 years of age	24-h dietary recall	Qualitative	1 day	Household	*N* = 736
74	Jones et al. (2014) (105)	To explore relationship and identify determinants between production diversity of household farms and HDDS	Secondary analysis of Cross-sectional survey	Malawi	Households	24-h dietary recall 7-d dietary recall -FFQ	Quantitative	1 day	Household	*N* = 6,623
75	Nkonde et al. (2021) (106)	To determine effect of agricultural diversification on household dietary diversity	Secondary analysis of Longitudinal survey	Zambia	Households with children below 5 years of age	24-h dietary recall	Qualitative	1 day	Household	*N* = 7,934
76	Khumalo and Sibanda (2019) (107)	To determine the contribution of agriculture in food security status of households	Cross sectional survey	South Africa	Households	24-h dietary recall Open based and list-based questionnaire 4-w dietary recall	Quantitative	1 day	Household	*N* = 208
77	Cordero-Ahiman et al. (2021) (5)	To analyze different factors determining HDDS	Cross sectional survey	Ecuador	Households	7-d dietary recall	Qualitative	1 day	Household	*N* = 383
78	Roba et al. (2019) (108)	To assess different indicators of household and individual food security status	Longitudinal survey	Ethiopia	Households	24-h dietary recall 7-d dietary recall 1-m dietary recall -FFQ	Qualitative	Twice (before and after harvest season)	Household	*N* = 800
79	Hirvonen et al. (2016) (109)	To assess role of seasonality on sources, intake of energy (*per capita*), and HDDS	Cross sectional survey	Ethiopia	Households	4-d dietary recall	Quantitative	Twice a week in every month for 1 year	Household	*N* = 27,835
80	Ngema et al. (2018) (110)	To assess the determinants of food security status of households	Cross sectional survey	South Africa	Households	24-h dietary recall	Qualitative and Quantitative	1 day	Household	*N* = 495

### 3.2 Critical appraisal of dietary data collection methodology

For data collection, 78 of the 80 studies employed well-trained interviewers to deliver face-to-face interviews to respondents, while two studies employed self-administered recalls. Three studies reported data collection through tablet-based surveys (19, 36, 45). Only one study, among the 80, used computer-assisted telephone interviewing in addition to interviewer-assisted face-to-face recall (19). All food items in dietary recalls were classified into major food groups as defined in the MDD-W, IYCF-MDD, and HDDS guidelines. Traditional and mixed foods, such as chicken curry and pizza, were disaggregated into respective ingredients and then included in their relevant food groups. The diet diversity score was then calculated by adding the total number of food groups consumed by an individual or the household in a 24-h period. Interviewers were required to attend training sessions on the study objective, data collection procedure, sampling method, ethical issues, data entry, and data management before traveling into the field in almost all the 78 studies. In two studies, employing self-administered recalls (75, 76), trained professionals were required at later stages to assess dietary data from forms. It should be highlighted that, unlike most studies, neither of these two kinds of research were undertaken in resource-poor settings. In the context of recalls, 67 studies employed 24-h recalls, three studies employed 7-d recall, five studies employed both 24-h and 7-d recall, and the remaining five studies employed 48-h recall, 4-d recall, 30-d recall, 1-y recall, and both 24-h and 4-w recall, respectively. The recalls were administered using list-based, open recall-based, or food frequency questionnaires. Although quantitative recalls can be challenging, especially in settings with low literacy rate, recalls practiced in 28 studies were quantitative, 33 were qualitative, five were semi-quantitative, three were both quantitative and qualitative, and 11 studies did not report on the type of recall. Dietary data was collected once in 52 studies, twice in 15 studies, and more than twice in the remaining studies. Data was collected from respondent’s household in 68 studies, health care facilities in 10 studies, and universities in two studies.

Among different methodologies, although there is no fixed gold standard diet evaluation method, the quantitative 24-h recall has been frequently employed in variety of applications such as describing intakes, examining associations, and evaluating the effects of interventions. Nevertheless, we cannot rule out the possibility of recall bias since retrospective methods tend to underestimate or overestimate actual food consumption for various reasons, including forgetfulness (111). Workshops for interviewers, including classroom training and fieldwork are required for the collection of high-quality data with minimal bias (45). Three studies reviewed in this paper evaluate the validity of different methodologies used for data collection (19, 45, 54). Hanley-Cook et al. (45) assessed the relative validation of qualitative list-based and open recall-based methods with reference to weighed food records. It was discovered that in these three countries (Cambodia, Ethiopia, and Zambia), both list-based and open recall-based methods were prone to misreport consumption of certain food groups. Reporting of food items that were not consumed in sufficient quantity, i.e., less than 15 grams for MDD-W, resulted in overreporting for both methods by 10%. These results were consistent with the findings of the second validation study conducted in India and Bangladesh (54), in which they assessed validation of qualitative list-based with reference to quantitative open recall-based methodology. The third validation study evaluated the performance of computer-assisted telephone interviewing (CATI) for collecting dietary data from African women in large-scale studies (19). The findings of this study revealed that switching from traditional in-person interviews increased the diet diversity scores by 11–14% in some indicators. This discrepancy could be the consequence of sensitive probes, which may unveil unfavorable information about the responder. The responses demonstrated a significant social-desirability bias, which can be mitigated by changing the mode of data collection.

## 4 Discussion

The review has attempted to describe all methodological aspects applied in diet diversity indicator studies, to critically assess the limitations of traditional methodology. According to findings of the review, most studies have employed interviewer-administered 24-h recalls to assess dietary diversity. However, every method possesses constraints that affect data collection and thus undermine conclusions of the research, especially if reported errors are not addressed to the maximum extent possible, using appropriate tool selection. Since they rely on memory and social perception of questions asked, major drawbacks include recall (51) and social-desirability bias (19). Furthermore, traditional methodology necessitates a significant amount of effort on part of the interviewer to probe and transcribe the respondent’s dietary intake, which comes with a high chance of errors and time-related costs (18). Technology adaptation has resulted in notable changes in dietary assessment methodologies, all of which have a favorable effect on cost, respondent-researcher workload, efficiency of data collection, coding and processing of dietary intakes, response rates, and the consistency of assessment measures (112). Since personal digital assistants (PDAs), tape recorders, scan- and sensor-based technologies have all become outdated, and all operations of web-based or computer-based platforms/ software can now be performed on smartphones, dietary evaluation via a smartphone-based application has a great potential (28). According to prior studies, smartphones are convenient, easy to use, and handy, thus preferred over conventional methodologies for recording dietary data (24). Additionally, smartphones possess the ability to overcome the shortcomings of conventional methodologies (113).

### 4.1 Respondent bias

#### 4.1.1 Recall

Dietary recalls ask respondents to remember and report all foods and beverages consumed in a specific time period, usually the preceding day’s 24 h. Dietary recalls are conducted without prior notice, eliminating the risk of reactivity (18). The use of a local interviewer to administer recall minimizes the literacy barrier and aids recall. However, many respondents have trouble distinguishing between what they consume habitually and what they ate the day before, leaving the door open to omissions and intrusions (114). The human ability to recall events fades over time, beginning within an hour after the meal consumption (115). It can be deduced that longer the recall period, greater is the bias (60). Furthermore, recalling foods eaten away from home is equally dependent on memory, which may reduce the validity of dietary recalls (37). Recall accuracy can be enhanced if executed several times over 24 h, hence minimizing the intrusion rate by shifting to a record-like approach from the recall approach (116). This can be accomplished by using a smartphone application that, due to its portability, can be always carried around by respondents and collects real-time self-administered dietary data on foods consumed via digital recording rather than through paper questionnaires. This will reduce the amount of effort and time required to fill out and decipher conventional forms in 24-h recall interviews, while increasing respondent motivation to record meals (28).

#### 4.1.2 Social-desirability

Social-desirability bias is the tendency of respondents to answer questions in a way they hope will be considered favorable by others (117). Generally, when the survey process is more socialized, respondents are more likely to give answers that are considered desirable by society (19). In dietary surveys, the bias can appear as over-reporting of “healthy diets” and under-reporting of “unhealthy diets.” Additionally, biases based on the sex of the interviewer are becoming more prevalent in the developing world. In one of the MDD-W studies, evidence was found that male interviewers were more likely to record lower diet diversity scores than female interviewers (19). At the point, when respondents are unsure about the interviewer’s probable response, or when the noting cycle does not include any relationship with others, the responses are based more on what respondents actually know or consume (118). The main cause of social-desirability bias, such as the presence of an acquaintance or interactions with the interviewer (44), can be avoided by switching from current traditional practices to technology-based methodologies. By ensuring respondent’s privacy, a smartphone app that allows them to record their dietary data without engaging in face-to-face interactions, by logging into their personal account, could help reduce social-desirability and sex-bias. Such biases in data recording are well documented, but the link between them and data collection methodology needs to be investigated further.

### 4.2 Interviewer training and burden

In-person interviews using traditional list-based or open recall-based questionnaires have their own set of functional benefits and drawbacks. The list-based methodology demands less interviewer capacity and training time; nonetheless, its implementation can be more time-consuming and prone to food misclassification, particularly for foods taken in little amounts (54, 119). For example, in a study conducted in India, milk added to tea, and onions or tomatoes added in mixed dishes were not identified by the list-based method (54). On the other hand, an open recall-based methodology can provide a more accurate and comprehensive recall of all food items consumed; however, it requires additional training and more skilled enumerators who have a working knowledge of local foods and recipes (120). In most of the studies, workshops on training and confidence-building were required during the preparatory phase to ensure precise and effective data collection. Following the collection of dietary information, incomplete columns were cross-checked, and paper questionnaires were meticulously numbered to preserve the record and privacy of respondents (44). Moreover, to ensure consistency, educated local personnel were required to develop questionnaires first in English, followed by a translation in local language, and finally back to English (44, 46, 60). In one study (45), the interviewers accompanied the respondents to measure the portion of foods consumed away from the household. To enhance interviewer confidence and assess the validity of data collection, some studies conducted small pilot surveys prior to the actual surveys. All these factors together add up to a significant increase in interviewer effort and time to collect the data. An interviewer-administered 24-h dietary recall via the ‘Automated Multiple-Pass Method’ (AMPM) can take 45–60 min in completion (121), increasing both respondent and interviewer burden. On the other hand, smartphone applications that ask structured questions about date/time, occasion of consumption, food name, constituent ingredients, portion size or number of servings, and where the meal was prepared or consumed, would not only reduce the interviewer’s workload, but also allow respondents to track their meals in their own time. Dietary planning is predominantly the duty of women in resource-poor settings. As a result, male interviewers can be less knowledgeable about the constituents of mixed dishes (19). A robust database containing the nutritional content of cooked and uncooked local foods linked with the application might reduce the labor involved in data collection, coding, analysis and provide the results at same time. This will result in a decrease in the dietary data’s reliance on the interviewer’s skill and ability. Prior studies have found a high level of agreement between traditional and modern approaches, with the latter being preferred by a majority of participants.

### 4.3 Cost–time constraints

In the field of dietary assessment, there is increasing pressure to enhance the accuracy, while lowering the data collection and processing cost involved in traditional methodologies (122). Training and data collection, which involves interviewing, coding, processing, and quality control, demands a significant amount of cost, and time during the research process. Dietary assessment studies commonly adopt technology to reduce the cost and complexity involved in collecting and processing dietary intake data (18). A study comparing different sampling methods among wine consumers claimed that the cost of a face-to-face survey was 2–2.5 times higher than the online surveys (123). In Kenya, while comparing the strengths and limitations of CATI with reference to face-to-face interviews, it was revealed that the former was determined to cost 5 US$ per survey and the latter was determined to cost 16 US$ per survey (19). Recently, a large number of 24-h recalls, and FFQ are being administered via modern technologies pertaining to lower costs (18). Furthermore, the primary disadvantage in the majority of the diet diversity indicator studies assessed was single-day data collection and limited sample sizes, which can be suppressed by smartphone applications, since no significant supplementary cost is required to expand the number of entries or participants. Researchers leading the development of ‘Automated Self-Administered 24-h Recall’ (ASA24) pointed out that research opportunities may arise from significant cost savings provided by newer technologies when compared to the equivalent quality of data (124).

Although most of the studies reviewed in this paper have not mentioned about how long the interviews took, studies conducted in Ethiopia (44) and Lebanon (58) revealed that interviews lasted an average of 30-min and 45-min, respectively. Longer interviews can be a demotivating element for respondents taking part in nutritional surveys. Respondents who are preoccupied with their work, may systematically disregard traditional time-consuming surveys and prefer smartphones over them. Smartphone applications can help in speeding up the data collection and analysis process. ‘My Meal Mate,’ (24) a weight-loss smartphone app, took an average of 7 min to record a meal, compared to 8.5 min for ‘DietMatePro’ (125) and 5 min for the ‘Wellnavi’ Personal Digital Assistant (PDA) device (126). Respondents reported spending an average of 22 min per day using the ‘My Meal Mate’ smartphone application for recording meals, which is comparable to a 24-h recall. However, the amount of time spent manually coding the data collected in the traditional method is far longer than with the smartphone application, which does not require any additional coding effort.

Also, the present situation of a novel virus, COVID-19, which spreads by encountering droplets of infected fluid (127), respondents might not be interested in participating in dietary surveys involving face-to-face interactions. A recent review centered on the efficiency and quality of data collection of studies during the COVID-19 pandemic revealed that 92% of studies collected data through web-based or app-based surveys (128).

Despite increasing popularity and ownership, smartphones are still not universal and have some limitations. There were legitimate concerns that new technology acceptance would be low among various population segments (even with access), notably among those who were not technologically skilled or knowledgeable (129). Prior research has demonstrated that respondents who were not using mobile devices, stated that they will not participate in a survey that does not allow them to maintain a paper diary, as an alternative to the technology-based approach (129). Switching from traditional methods may necessitate respondent training on tool usability and might increase their workload in absence of the interviewer (130). ‘Response fatigue’ is associated with self-administered respondent recordings, that last more than four consecutive days (131). Therefore, as the week progresses, the accuracy of dietary data being recorded by the respondent might more likely be compromised. Moreover, it has been acknowledged that well-off, educated, and knowledgeable respondents tend to make a major proportion of technology-based surveys (132). Being more informative, they can have better dietary habits and diet diversity scores. Consequently, collecting data via smartphone applications can be biased if the population that can be reached via smartphone differs from the general population (non-coverage bias) or if the responding population differs from the non-responding population (non-response bias) (19).

However, the collection of data by mobile phones has evolved over time, from a rarely used and frequently criticized method to a dominant mode of data collection all over the world (123). By reducing the duration involved in collecting and reporting food consumption data, while enhancing the quality by limiting misreporting errors, newer technologies have gained an edge over traditional methodology (132). Automated dietary assessment methods have the potential to reduce respondent and researcher burden while giving the flexibility of a prospective method in terms of food reporting (24). Even though the methodological features of smartphone applications and traditional methods might frequently overlap, smartphones have the potential to improve dietary assessment by allowing lesser respondent-researcher burden, more cost- and time-effective data collection, a wider geographic reach, and greater respondent acceptability.

## 5 Conclusion

The review has attempted to describe all methodological aspects implemented in MDD-W, IYCF-MDD, and HDDS studies to critically assess the limitations in traditional methodology and fill the gap with inventive smartphone application that works in tandem with technology and modernity. Traditional methods have inherent limitations, such as recall bias, social desirability bias, interviewer burden, and cost–time constraints, which impair data collection and thus undermine the research conclusions. Smartphone adaptation might result in notable changes in dietary assessment methodologies to make a favorable effect on cost, respondent-researcher workload, efficiency of data collection, coding and processing of dietary intakes, response rates, and the consistency of assessment measures. In conclusion, while the transition from conventional to smartphone applications is recommended for collecting dietary data, the relationship between the efficiency, effectiveness, and quality of data collection using both methodologies warrants further investigation.

## Author contributions

SM, EK, and MN: conceptualization. SM, CK, EK, and MN: methodology, formal analysis, and investigation. SM, CK, MN: data curation. SM, CK, and EK: writing–original draft preparation. CK, EK, and MN: writing–review and editing and supervision. MN: funding acquisition. All authors contributed to the article and approved the submitted version.
